# Effects of Personalized Messaging From a Social Media Influencer on Followers’ Step Counts: A Parallel-Group Randomized Controlled Trial

**DOI:** 10.7759/cureus.88184

**Published:** 2025-07-17

**Authors:** Ryosuke Shigematsu, Takumu Ichikawa, Yoshitake Oshima, Hiroyuki Sasai

**Affiliations:** 1 School of Health and Sport Sciences, Chukyo University, Toyota, JPN; 2 Course for Health and Physical Education, Faculty of Education, Mie University, Tsu, JPN; 3 Department of Physical Education, Kyoto University of Education, Kyoto, JPN; 4 Research Team for Promoting Independence and Mental Health, Tokyo Metropolitan Institute for Geriatrics and Gerontology, Tokyo, JPN

**Keywords:** exercise, online social networking, social media, text messaging, young adult

## Abstract

Introduction: Physical activity during adolescence is crucial for long-term health; however, many young individuals lack adequate activity levels. Social networking services (SNS) and influencers have shown promise in shaping behaviors; however, their potential to promote physical activity remains underexplored, especially when influencers are not fitness-related. This study investigated whether personalized messages from a non-fitness influencer could increase followers' physical activity.

Methods: A parallel-group randomized controlled trial was conducted for four weeks in December 2019 among followers of a non-fitness influencer of a Japanese idol group. Participants were assigned to either an individual message group (personalized messages) or an automated message group (standardized messages). Step counts were tracked via a smartphone application. Statistical analyses assessed the differences in daily steps and goal achievement (baseline average plus 1,000 steps) between the groups.

Results: With a total of 120 participants, the mean difference in step count during Weeks 3 and 4 was significantly greater in the individual message group than in the automated message group. Goal attainment was also greater in the individual message group than in the automated message group at Weeks 2-4.

Conclusion: Personalized messages from non-fitness influencers effectively increased physical activity, suggesting a scalable strategy for promoting health in young populations through SNS platforms.

## Introduction

The health and well-being benefits of physical activity in children and adolescents are widely recognized [1]. Low levels of physical activity during this period are associated with continued inactivity in middle age [2] and an increased risk of lifestyle-related diseases [3-5]. Therefore, it is critical to promote an active lifestyle during adolescence.

Adolescents extensively use social networking services (SNS) in their daily lives [6]. Recognizing that SNS influence the thoughts and behaviors of many people, especially adolescents, commercial companies often engage with influencers and celebrities to promote products and services [7]. From a public health perspective, leveraging purchase motivation has been recognized as a strategy to promote healthier lifestyle choices [8]. Recent studies have demonstrated the effectiveness of influencers in promoting behaviors such as smoking cessation [9], alcohol moderation [10], and healthy eating [11]. These findings highlight the potential of influencers to address public health challenges innovatively.

Several studies have explored the role of influencers in promoting physical activity, primarily focusing on exercise and fitness [12, 13]. While these influencers may motivate their followers to exercise and maintain high levels of physical activity, the individuals who benefit the most from increased activity are not their followers but those who are physically inactive.

A systematic review indicated that face-to-face personalized interventions are effective in promoting physical activity among those who are not necessarily active [14]. This suggests that influencers from non-fitness fields could effectively engage those who are not already interested in exercise or do not have regular routines when providing personalized interventions. Therefore, targeting these individuals plays a vital role in extending the reach of public health efforts. Although fitness influencers have been widely studied, the strategic use of non-fitness influencers to reach physically inactive populations has received little attention and remains an unexplored opportunity. The purpose of this study was to investigate whether a non-fitness influencer can increase their followers’ physical activity levels. We hypothesized that personalized encouragement from non-fitness influencers would lead to increased physical activity among followers.

## Materials and methods

Study design

This study was conducted as a parallel-group, non-blinded, randomized controlled trial. Participants were assigned to either an individual message group, in which personalized communications were provided, or an automated message group, in which standardized messages were automatically sent to each participant regardless of their physical activity. After consenting to participate in the study, each participant's baseline physical activity was measured over a seven-day period beginning on November 20, 2019, and the intervention was delivered over a 30-day period beginning on November 28 and ending on December 27, 2019. All measurements and interventions were conducted online, allowing participants to complete the study from home, work, or school, whereas the researchers conducted the study remotely.

This study was conducted after review and approval by the Research Ethics Review Committee of the Faculty of Education, Mie University, Tsu, Japan (approval no. 2019-8). This study was registered in the Clinical Trials Registry System (UMIN000042411; https://center6.umin.ac.jp/cgi-open-bin/ctr/ctr_view.cgi?recptno=R000046381). The trial is described in accordance with the Consolidated Standards of Reporting Trials (CONSORT) statement [15] and Consensus on Exercise Reporting Template (CERT) [16] to ensure detailed reporting.

Influencer

This study focused on a male influencer in his 20s with an academic background in education and public health who actively tweeted about a Japanese female idol group. As of 2019, the group's music catalog included 35 singles and 22 compilation albums. The influencer enjoyed communicating with his fellow idol group fans on X (formerly Twitter, Meta Platforms, Menlo Park, CA). Although he was a non-fitness influencer, the researchers met him by chance and invited him to collaborate as an influencer.

The influencer created an X account in December 2016 and gained more than 10,500 followers by January 2018 through tweets about the idol group. However, this account was deleted in the same month owing to a hacking attempt. In February 2018, he launched a new account, regaining approximately 2,000 original followers and reaching over 3,000 followers within three months. Between June 1 and August 30, 2018, the daily tweet impressions averaged 59,209, with an engagement rate of 4.2%, before the account was deleted in October 2018, following another hacking attempt. A third account was created in October 2018, which accepted followers who had previously interacted privately with the influencer. As of March 2019, his most recent account had 154 followers, approximately half of whom met the influencer at the idol group’s performances. Owing to the retrospective nature of data collection, we were unable to obtain specific details about these in-person interactions.

Participants

The target population was defined as “followers of an influencer who had become famous for a topic other than physical activity and exercise.” The influencer invited all 154 followers to participate in the study via direct X messages approximately two weeks prior to the baseline measurement. The participants were required to be at least 18 years old and have no disabilities or medical conditions that would make walking difficult.

**Figure 1:**
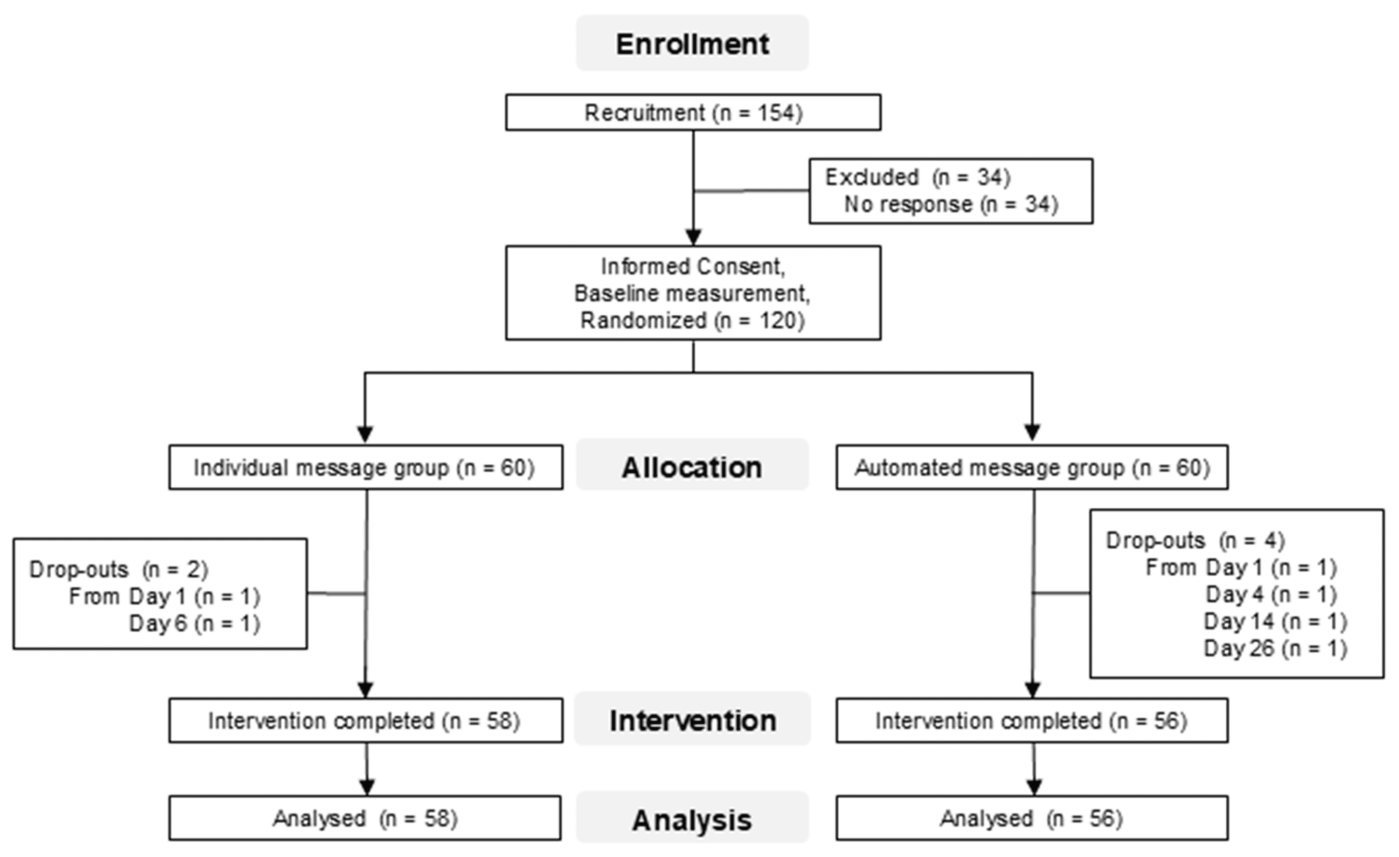
Participants flow diagram

To account for variability in physical activity due to weather and temperature [17], participants were stratified into six Japanese regions of residence. They were then further stratified by employment status (worker/student) within each region and randomly assigned (1:1) using the RAND function in Microsoft Excel (Microsoft Corp., Redmond, WA) after confirming the number of enrolled participants. Group assignments were immediately disclosed to participants, researchers, and the influencer, with no third-party concealment implemented. Dropouts were defined as those who did not report their daily step count in a given week. Those who did not drop out were defined as those who completed the protocol (per protocol) (Figure 1).

Intervention

Communication

The individual message group received personalized daily messages from the influencer, whereas the automated message group received standardized preprogrammed messages. For this study, the influencer used LINE (LINE Corporation, Tokyo, Japan, https://line.me/en/) for the individual message group and LINE@ for the automated message group. Both applications, which are popular in Japan, offer similar functions to WhatsApp Messenger and Viber. Note that LINE@ has since been renamed to "LINE Yahoo for Business," but this name change, a part of the company’s business strategy, had no impact on the intervention or data collection. Participants were instructed to send their daily step counts via LINE or LINE@ by 9 a.m. the following day. Those who did not report received a reminder at that time.

Behavior Change Messages

**Figure 2:**
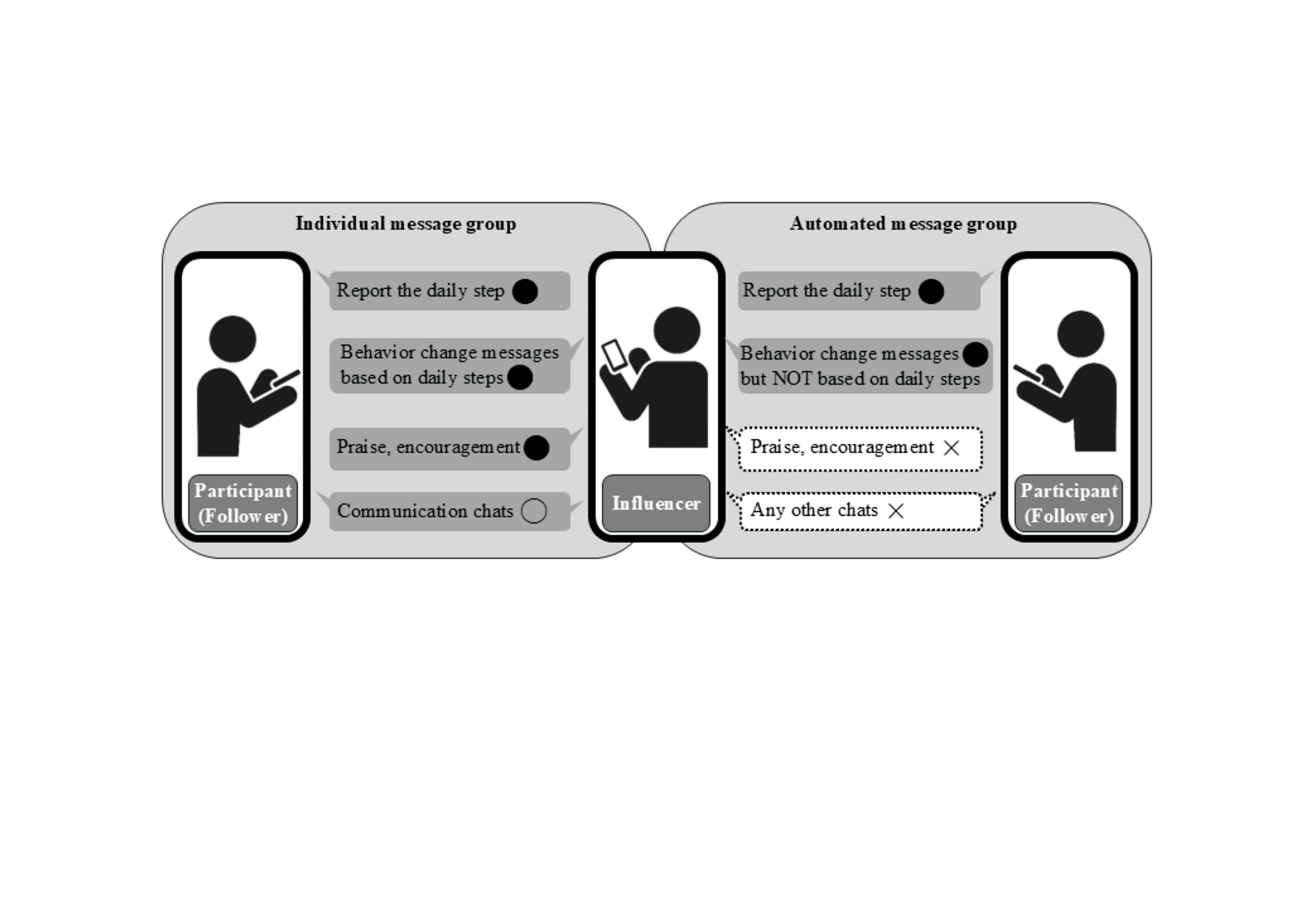
Communication of the intervention in each group ● Everyday; ○ On-demand; × Never This figure has been created by the authors.

Behavioral change messages were the same for both groups. The messages to increase physical activity were based on 15 behavioral change theories [18], such as social cognitive theory, decision-making theory, and the relapse prevention model. One message based on each theory was sent on Days 1-15 of the intervention, and the same theory was used on Days 16-30, but with different examples. The name of the theory underlying the message was also provided to increase credibility (Figure 2, Appendix A). In this study, personalized messages were often tailored to explore the reasons participants failed to meet their goals and then encouraged them with specific advice on how to overcome those barriers. In some cases, the messages were modified to reassure participants not to feel discouraged about missing a goal, reminding them instead that achieving it the next day would still be meaningful. The behavioral change theory used that day was sometimes explained again in a more casual tone within the message.

In the individual message group, the beginning of the message changed depending on the participant’s goal achievement. There were three messages: (a) For participants who walked more than 1,000 steps above the baseline, the message was, “Congratulations on achieving your goal! Let's walk again so that you can achieve your goal today”, (b) for participants who did not achieve the goal but did not fall below the baseline steps, “You were very close to your goal,” and (c) for participants who fell below the baseline steps, “Let's be conscious of walking more today than yesterday so that you can achieve your goal.”

The influencer continuously measured the number of steps taken during the study period and occasionally reported the results to the participants. When asked about the progress of other participants (number of steps and reporting status), the influencer responded. However, the personal information of the participants was withheld to maintain privacy.

All participants in the automated message group received all three behavioral messages (a, b, and c), regardless of whether they achieved their goals. The messages did not include praise. The messages in both groups were sent once daily by 9 a.m. the following morning.

Measurements

Primary Outcome

The primary outcome was the number of steps taken per week during the intervention period, measured using the Pacer app (Pacer Health, San Francisco, CA, https://www.mypacer.com/) on the participants' smartphones. The participants sent screenshots of their daily step counts to the influencer via LINE or LINE@. After the intervention period, each participant was asked to send a comma-separated values (CSV) file containing 30 days of step data, which was saved in the Pacer app and analyzed. As a preliminary investigation, three individuals (two males and one female, all aged 22 and not part of the main study) performed free walking. The actual step count was 1,167 ± 492 steps, while the Pacer app showed 1,163 ± 495 steps. The mean difference was 4.0 ± 6.3 steps (0.6% ± 0.9%), indicating high measurement accuracy.

The number of steps taken each day was totaled for seven days, and the average was used as the number of steps taken that week. It was calculated and compared with the number of steps taken at baseline (Week 0). Days 1-7 were defined as Week 1, Days 8-14 as Week 2, Days 15-21 as Week 3, and Days 22-28 as Week 4. Data from the last two days (29^th^ and 30^th^) were ignored to diminish the potential upward bias from higher activity as a “last spurt,” although the procedure was not communicated to the participants.

Although the amount of time per day that the device was worn (wearing time) was not specified, the participants were asked to wear the device during the same time period as the baseline measurement. The average time spent carrying a smartphone that was saved in the Pacer app was calculated for each week.

Secondary Outcomes

Secondary outcomes included goal attainment, adherence, and serious adverse events. Goal attainment was defined as the percentage of intervention days on which participants met their daily step goal set at their baseline average plus 1,000 steps [19]. Adherence was assessed by the number of days participants reported their step counts. Serious adverse events, such as injuries and illnesses requiring hospitalization, death from any cause, or life-threatening events regardless of their relation to walking, were reported daily via LINE or LINE@ during the study period. When such events were reported, participants were to be asked to cease the protocol and focus on treatment or recovery.

Statistical Analyses

Descriptive statistics were examined to check the quality of the data. The Shapiro-Wilk test was used to determine whether the data followed a normal distribution. The Welch t-test was used to compare the initial values of the number of steps between the dropout participants and the participants who completed the intervention, and the percentage of days on which the number of steps taken the previous day was reported the following day between the groups. The Mann-Whitney U test was used when the assumption of normality was not met.

A two-way analysis of variance (ANOVA) was conducted to assess differences in means across groups and over time. Age, sex, and employment status were used as covariates. If the interaction was significant, Tukey's multiple comparison test was performed for further analysis. The effect size was calculated as the generalized η^2^ (η^2^G). The rates of goal achievement and next-day responses between the groups were compared using the Mann-Whitney U test.

All analyses were conducted using a per-protocol approach to assess the potential benefit of the intervention among individuals who fully adhered to the protocol. In cases of missing data, no imputation was performed prior to analysis. Jamovi (ver. 2.3.28, The jamovi project (2023). Retrieved from https://www.jamovi.org), built on R (ver. 4.1, (The R Core Team, R Foundation for Statistical Computing, Vienna, Austria), was used for all statistical analyses. Statistical significance was set at p < 0.05.

## Results

Participants

After calling 154 followers, 120 agreed to participate in the study (Figure 1). The number of participants, age, sex, employment status, residential region, and number of steps taken per day at baseline were compared between the two groups (Table 1). The smartphones used included an iPhone 6s or later (Apple Inc., Cupertino, CA, USA) with a three-axis gyroscope and an accelerometer (Core Motion, Apple Developer Documentation, Apple Inc.) (n = 116), AQUOS (Sharp Corporation, Osaka, Japan) (n = 2), and Xperia (Sony Mobile Communications Inc., Tokyo, Japan) (n = 2).

**Table 1:** Participants' characteristics at baseline measurement Data are shown in mean ± standard deviation.

Characteristic	Total			Individual message group			Automated message group		
Age (y)	19.7	±	1.5	19.8	±	1.5	19.6	±	1.5
Women (%)	22%			21%			23%		
Occupation									
Worker (%)	80%			78%			82%		
Student (%)	20%			22%			18%		
Residential region									
Hokkaido/Tohoku (n)	9			4			5		
Kanto (n)	44			22			22		
Chubu (n)	25			13			12		
Kinki (n)	28			14			14		
Chugoku/Shikoku (n)	7			3			4		
Kyushu (n)	7			4			3		
Daily steps (steps)	6,333	±	3,160	6,450	±	3,127	6,212	±	3,219

Two participants in the individual message group and four in the automated message group dropped out during the intervention period due to the absence of step count reports (Figure 1). The six dropouts had a baseline step count of 6,193 ± 4,507 steps, which was not significantly different from the 6,479 ± 6,173 steps of the 114 participants who completed the intervention (p = 0.874).　

Primary outcome

**Figure 3:**
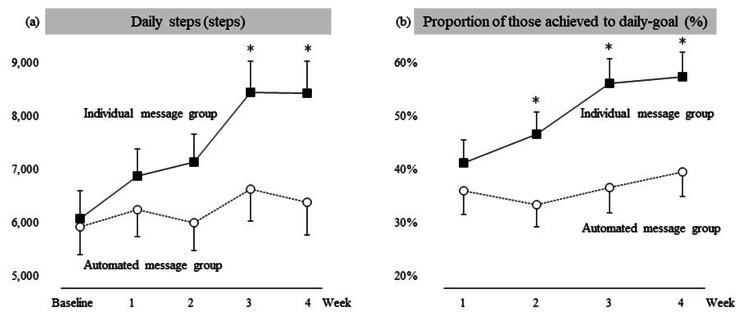
Changes in mean daily steps (a) and proportion of those achieved to daily goal (b) by group and week. Black squares and white circles indicate means in each week in each group. Vertical lines show standard errors of the mean. Interactions of week by group were significant (p = 0.01). Asterisks indicate statistical significance between groups. Age, sex, and employment status were used as covariates.

A two-factor ANOVA showed that the interaction between group and time was significant in their daily step count (F = 3.550, p = 0.01, η^2^G = 0.007). The mean increase in step count from baseline during Weeks 3 and 4 was significantly greater in the individual message group than in the automated message group (mean difference between the groups: 1,656 (95% CI: 659-2,652); 1,945 (95% CI: 796-3,094), respectively) (Figure 3).

Although wearing time was not included as a covariate in the analysis, it did not differ significantly between groups during the intervention period. The mean and 95% CI were 461 (429-493) min/day in the individual message group and 428 (389-467) min/day in the automated message group. When the number of steps divided by wearing time was compared between the two groups, the interaction was found to be statistically significant. A marginally significant post hoc test showed greater Week 4 step counts in the individual message group than in the automated group (17.7 and 14.0 steps/min, respectively; p = 0.052).

Secondary outcomes

When goal attainment was calculated for each group on a weekly basis and analyzed with a two-way ANOVA, the interaction was statistically significant (F = 3.675, p = 0.013, η^2^G = 0.012). Goal attainment in the individual message group was also statistically greater than that in the automated message group at Weeks 2-4 (difference between the groups: 13% (95% CI: 4%-22%); 19% (95% CI: 9%-29%); 18% (8%-28%), respectively).

During the 30-day intervention, next-day responses were perfect in the individual group (100% (interquartile range (IQR): 100-100)) and slightly lower in the automated group (100% (IQR: 93-100); W = 957, p < 0.001)). No serious adverse events occurred during the study period.

## Discussion

The individual message group demonstrated an increase of approximately 1,800 daily steps during Weeks 3 and 4, along with improved goal attainment rates, which were also better than those in the automated message group. These results highlight the potential of personalized online support from non-fitness influencers to effectively enhance physical activity, thus supporting the study hypothesis.

Adolescence represents a critical period when attitudes toward exercise and fitness levels significantly influence long-term physical activity and disease risk [20, 21]. This study is significant because it increased the amount of physical activity among young people, and it is possible that this will have a positive impact on their future lives and lifestyles.

The smartphone-based intervention proved effective in increasing physical activity and reducing sedentary behavior, which is consistent with previous findings [22]. Such methods have gained importance following the COVID-19 pandemic, which has accelerated the adoption of mobile health interventions [23]. Mobile interventions offer broader reach and accessibility than traditional tools such as letters or phone calls.

The study’s goal-setting strategy of walking an additional 1,000 steps per day was both realistic and sustainable, equating to 10-15 minutes of walking and enhancing self-efficacy, which is a key driver of increased physical activity in youth [24]. During Weeks 3 and 4, the study participants exceeded the average step count for Japanese adults in their twenties (7,332 steps) and met the Health Japan 21 (Third Phase) target of 8,000 steps [25].

Behavior change techniques such as self-monitoring, goal setting, and positive reinforcement remain effective despite being developed before the smartphone era [23]. This program incorporated these strategies while intentionally excluding peer-sharing interventions, which can increase physical activity [26] but may lead to negative social comparisons on platforms such as Facebook [23].

The lack of improvement in the automated message group contrasts with prior research showing that personalized and frequent messages can significantly increase physical activity [27]. This difference may stem from the study's use of a single, non-customized message without personalized communication, whereas previous studies employed more tailored and frequent messaging strategies.

The strong emotional connections between followers and influencers likely contributed to the success of the intervention, which should be measured in future studies. Participants often continued to follow the influencer despite account changes, indicating loyalty that may have enhanced their motivation [28, 29]. Furthermore, the influencers’ public health expertise likely enhanced the program's effectiveness, aligning with evidence that peer-led interventions without relevant expertise are less successful [30].

However, several challenges warrant consideration. The concept of “fitspiration” raises concerns about excessive exercise [31], although participants’ activity levels in this study remained within safe ranges, as evidenced by an upper 95% CI of 9,600 steps in Week 4. Additionally, creating individual messages required several hours of effort from the influencer daily, underscoring the need for sustainable solutions. This suggests that the intervention could be scalable if artificial intelligence (AI) learns the style of the influencer’s words and thoughts and utilizes it. Future research should examine the cost-effectiveness of this approach and explore how it could be integrated into existing health programs.

This study had several strengths. It successfully engaged a previously unexplored population by leveraging influencers not connected to fitness or health, enabling a geographically flexible, low-risk, mobile intervention. The program yielded significant outcomes without serious adverse effects. Despite a small effect size, it showed strong statistical power in daily step counts (80.2%; η²G = 0.012, f = 0.11, α = 0.05, n = 114).

This study has several limitations. It only assessed step counts without examining the physical activity intensity or type, thus limiting the evaluation of overall fitness improvements. The intervention began two days after the baseline assessment. Although no formal feedback was provided, participants could view their own baseline step counts in the app, which may have influenced their behavior. Some participants’ prior face-to-face interactions with the influencer may have strengthened the intervention’s effectiveness but reduced its generalizability to purely online relationships. Additionally, the non-participation rate of 22% (34/154) could have introduced selection bias. Finally, the long-term sustainability of the intervention's effectiveness remains unexamined, leaving opportunities for future research.

## Conclusions

The online personalized message intervention by a non-fitness influencer effectively increased daily step counts and goal attainment among followers. Healthcare professionals can adopt this innovative approach while incorporating AI to reduce influencers’ workload.

To the best of our knowledge, no prior study has demonstrated the effectiveness of personalized online messages from non-fitness influencers in promoting physical activity. Future research should refine the intervention methods, explore influencer and follower characteristics, and evaluate the long-term sustainability of these effects.

## Appendices

Appendix A

A message based on each theory was sent on Days 1-15 of the intervention, and the same theory was used on Days 16-30, but with different examples. The 15 behavioral change theories are: (1) increasing knowledge, (2) being aware of risks, (3) caring about consequences to others, (4) comprehending benefits, (5) increasing healthy opportunities, (6) substituting alternatives, (7) enlisting social support, (8) rewarding yourself, (9) committing yourself, (10) reminding yourself, (11) learning theory, (12) decision-making theory, (13) behavioral choice theory, (14) social cognitive theory, and (15) relapse prevention model.

[Example message for the individual message group on Day 5] To those who have achieved their goals: Congratulations on achieving your goal! Let's walk again so that you can achieve your goal today. From now on, it might be interesting to try a slightly longer route home and see if you can discover something new. Let's all do our best to reach your goals today as well. For those who were less than 1,000 steps from their goal: You were very close to your goal. From now on, it might be interesting to take a slightly longer route on your way home, and you might discover something new. Let's try our best to reach your goals today. For those who have more than 1,000 steps to go: Let's be conscious of walking more today than yesterday so that you can achieve your goal. On your way home today, it might be interesting to take a longer route and see what new things you can discover. Let's try to walk more than you did yesterday. Common message for each participant: Today, I sent a message based on a behavioral change technique relating to “increasing healthy opportunities.”

The following is an example of a message and subsequent chats with a participant on Day 5:

*   1:06 a.m.* Participant A: Tomorrow is the last day of the university exams.

*   1:23 a.m.* Influencer: Oh! Restarting tomorrow, I expect you to walk more!

(Example message for the automated message group on Day 5] Participants in this group received the same standardized message regardless of goal achievement. Each message combined all three behavioral components that were delivered individually in the individual message group. The content was identical across all participants.
